# Engineering apomixis in crops

**DOI:** 10.1007/s00122-023-04357-3

**Published:** 2023-05-18

**Authors:** Alexander Mahlandt, Dipesh Kumar Singh, Raphael Mercier

**Affiliations:** Department of Chromosome Biology, Max Planck Institute for Plant Breeding Research, Carl-von-Linné-Weg 10, Cologne, Germany

## Abstract

Apomixis is an asexual mode of reproduction through seeds where progeny are clones of the mother plants. Naturally apomictic modes of reproduction are found in hundreds of plant genera distributed across more than 30 plant families, but are absent in major crop plants. Apomixis has the potential to be a breakthrough technology by allowing the propagation through seed of any genotype, including F1 hybrids. Here, we have summarized the recent progress toward synthetic apomixis, where combining targeted modifications of both the meiosis and fertilization processes leads to the production of clonal seeds at high frequencies. Despite some remaining challenges, the technology has approached a level of maturity that allows its consideration for application in the field.

## The goal of engineered apomixis

In the last century, significant improvements in yield and other desirable crop traits were seen following the widespread adoption of hybrid crops, the product of F1 crosses between two high performing, divergent lines (Hochholdinger and Baldauf 2018). Hybrid vigor, or heterosis, is the observed higher performance of an F1 hybrid over that of either parent line; this performance is reduced following the random segregation of genetic information in subsequent generations. Utilization of hybrid vigor thus depends today on the production of F1 hybrids by crosses. Apomixis is a naturally occurring mode of reproduction in plants that forms seeds identical in genetic makeup to the maternal parent and represents an efficient means of clonal reproduction that could fix parental genotypes such as F1 hybrids. The occurrence of apomixis is, however, absent in modern crops and restricted to widely spread but majorly non-cultivated plant species. In this review we aim to summarize work to synthetically produce apomixis in economically relevant crop species by linking natural apomixis with engineering strategies, with a further outlook for its suitability in modern agriculture.

## Sexual reproduction in flowering plants

**Fig. 1:**
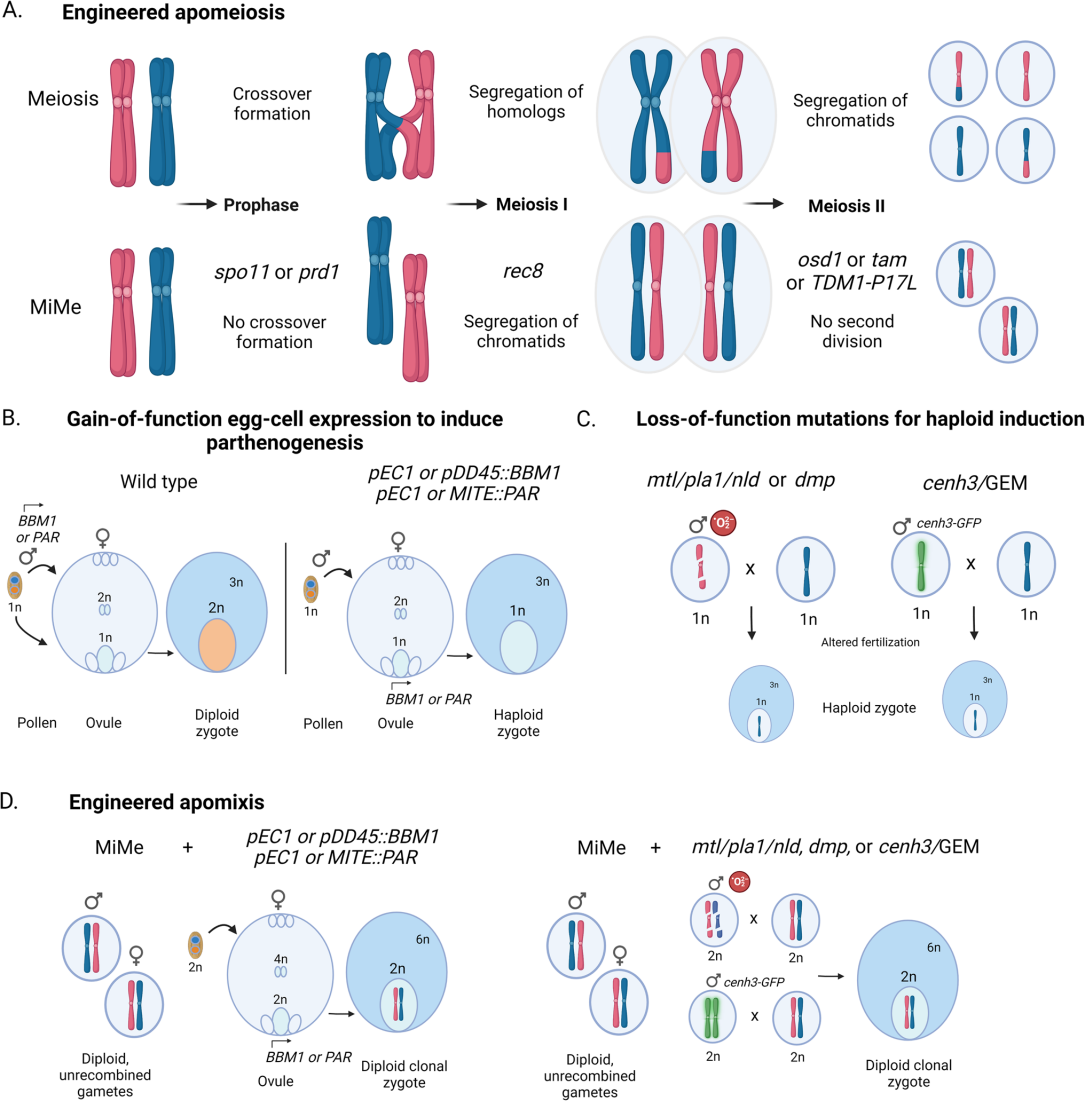
Engineering apomeiosis and parthenogenesis. Mutations in three meiotic genes (MiMe) alter crucial stages of meiosis to result in a mitotic-like division of chromosomes, mimicking and providing a tool to implement apomeiosis (**A**). Embryogenesis in many plants results from fertilization of the female-derived ovule and central cell by the male-derived pollen to give rise to a diploid zygote and triploid endosperm (**B**, left). Prior to fertilization, *BBM1* and *PAR* are expressed in the male gamete; redirecting their expression to the ovule can result in the formation of haploid zygotes (**B**, right). Alternatively, mutations in *MTL/PLA1/NLD, DMP,* or *CENH3* can hinder fertilization by disrupting one parental gamete contribution, and can produce haploid zygotes (**C**). By pairing MiMe with male expressed *BBM1/PAR* or mutations in *MTL/PLA1/NLD, DMP,* or *CENH3,* clonal progeny can be obtained that represent synthetic apomicts (**D**). Figure created with BioRender.com

Flowering plants have alternating phases, in which the sporophytic diploid phase (2n) alternates with the gametophytic haploid phase (n). Meiosis, occurring in both male and female organs, constitutes the transition from the sporophytic to the gametophytic phase. It produces haploid spores that divide mitotically to generate the male (pollen grain) and female (embryo sac). The gametophytic structures are composed of only a few cells and rely on the sporophyte for nourishment and development. In females, only one of the four spores survives and undergoes three mitotic divisions to form an eight-nuclei embryo sac. In the mature embryo sac, three cells are toward one side (micropylar, entry point of the pollen tube), the egg cell and two synergid cells, while three antipodal cells lie at the opposite side (chalazal end), while in between lies a double haploid (diploid) central cell that is produced by the fusion of two haploid cells (Skinner and Sundaresan 2018). In males, each of the four haploid cells survives and divides twice mitotically to generate a pollen grain that contains two sperm cells and one vegetative cell. At fertilization, the sperm cells are delivered by the pollen grain to the female gametophyte. One sperm cell fuses with the egg cell, resulting in a diploid embryo (Fig. 1B). The second sperm cell fuses with the diploid central cell, resulting in a triploid endosperm, the nourishing tissue of the embryo in the seed (Hafidh and Honys 2021).

## Natural apomicts

Meiosis and fertilization are thus the two key steps of the sexual life cycle in flowering plants. Some plants, however, bypass meiosis and fertilization to reproduce asexually through seeds, a mode of reproduction known as apomixis. Apomixis (gametophytic apomixis) has been documented in about 400 species (Asker and Jerling 1992; Carman 1997; Kellogg 1990; Majeský et al. 2017) and distributed across more than 30 families (Table 1), but the majority of the species belong to three families: Poaceae, Asteraceae and Rosaceae. Adventitious embryony is mostly found in Orchidaceae and Rutaceae citrus plants (Asker and Jerling 1992; Carman 1997; Hand and Koltunow 2014; Hojsgaard et al. 2014).

**Table 1:** Taxonomy of natural apomicts

Family	Example	Type of apomixis	References
Poaceae	Paspalum	Apospory	Brozova et al. (2019), Burson (1997), Carman (1997) and Ortiz et al. (2013)
Asteraceae	Hieracium	Apospory and diplospory	Koltunow et al. (2011) and Noyes (2007)
Rosaceae	Potentilla	Apospory	Brozova et al. (2019), Dickinson (2018), Dobeš et al. (2013), Majeský et al. (2017), Talent and Dickinson (2007)
Brassicaceae	Boechera	Diplospory	Böcher (1952), Dobes et al. (2004), Sharbel et al. (2005) and Voigt-Zielinski et al. (2012)
Ranunculaceae	Ranunculus	Apospory	Barke et al. (2020), Brozova et al. (2019), Carman (1997), Cosendai and Hörandl (2010) and Majeský et al. (2017)
Rutaceae	Citrus	Sporophytic	Nakano et al. (2013), Shimada et al. (2018), Wang et al. (2022) and Wang et al. (2017)
Orchidaceae	Rhomboda tokioi	Sporophytic	Teppner (1996), Xiao et al. (2021)
Melastomataceae	Miconia	Sporophytic, aposopy	Caetano et al. (2018), Caetano and Oliveira (2022), Viana et al. (2021)
Hypericaceae	Hypericum	Apospory, diplospory	Carman (1997), Galla et al. (2015)
Clusiaceae	Garcinia	Diplospory	Carman (1997), Pangsuban et al. (2009)
Adoxaceae	Sambucus, Fritillaria maximowiczii	Apospory	Carman (1997), Tong et al. (2022)
Amaranthaceae	Amaranthus palmeri, Beta	Diplospory	Carman (1997), Ribeiro et al. (2014)
Magnoliaceae	W. septentrionalis	Apospory	Qing-Wen et al. (2003)
Ochnaceae	Ochana	Diplospory, apospory	Carman (1997)
Plantaginaceae	Globularia	Apospory	Carman (1997)
Urticaceae	*Elatostema, D*orstenia, Boehmeria	Dipospory, apospory	Carman (1997), Firetti (2017) and Fu et al. (2017)
Bignoniaceae	Anemopaegma: A. acutifolium, A. arvense, A. glaucum and A. scabriusculum	Sporophytic	Firetti (2017), Firetti-Leggieri et al. (2013)
Amaryllidaceae	Allium, Habranthus, zephyranthes, Ornithogalum	Diplospory	Carman (1997)
Burmanniaceae	Burmannia	Diplospory	Carman (1997), Ernst (1909)
Taccaceae	Tacca (Schizocapsa)	Apospory	Carman (1997)
Balanophoraceae	Balanophora	Diplospory	Carman (1997), Gonzalez et al. (2019)
Cyrillaceae	Cliftonia	Apospory	Carman (1997)
Saururaceae	Houttuynia	Diplospory	Carman (1997)
Rhamnaceae	Pomaderris	Diplospory	Carman (1997), Chen et al. (2019)
Thymelaeaceae	Wikstroemia	Diplospory	Carman (1997)
Myrtaceae	Eugenia	Apospory	Carman (1997), Souza-Pérez and Speroni (2017)
Plumbaginaceae	Limonium	Diplospory	Carman (1997), Sáez and Rosselló (1996)
Polygonaceae	Atraphaxis	Apospory	Carman (1997)
Casuarinaceae	Casuarina	Diplospory	Carman (1997)
Betulaceae	Alnus	Diplospory	Carman (1997), Woodworth (1930)
Malpighiaceae	Hiptage	Apospory	Carman (1997)
Boraginaceae	Cordia	Apospory	Carman (1997)
Cucurbitaceae	Cucumis, Luffa, Maras, Momordica, Bryonia alba	Dipospory, apospory	Carman (1997), Novak and Mack (2000)
Araceae	Aglaonema	Apospory	Carman (1997)

Several types of natural apomixis have been defined, depending on the origin of the clonal embryo: gametophytic apomixis retains the development of an embryo sac and is further divided into diplospory and apospory, depending on the origin of this embryo sac (Koltunow and Grossniklaus 2003; Underwood and Mercier 2022). In diplospory, a modified female meiosis that resembles mitosis produces non-recombined, clonal diploid spores. This spore divides mitotically to form a mature embryo sac that cytologically appears similar to the wild type. The egg cell, which is thus diploid and clonal, enters embryogenesis without fertilization, a process known as parthenogenesis. In apospory, a somatic cell derived from the ovule develops directly into a diploid embryo sac. The presence of several embryo sacs or the absence of antipodal cells distinguishes apospory from other forms of apomixis (Conner and Ozias-Akins 2017; Koltunow 1994). Although each ovule frequently produces several aposporous embryo sacs, typically only one of these develops into an embryo by parthenogenesis. Another mode of apomixis, classified as sporophytic, is adventitious embryony, in which embryos develop directly from somatic cells of the ovule. Because sexual reproduction occurs in the same ovule in parallel to apomictic embryogenesis, polyembryonic seed development is very common in sporophytic apomixis.

On the basis of the penetrance of apomictic progeny formation, apomicts are classified into two groups: obligate apomicts and facultative apomicts. Obligate apomicts reproduce mostly through apomictic means, but a small fraction reproduces sexually, whereas facultative apomicts show the reverse (Asker and Jerling 1992; Mráz and Zdvořák, 2019).

A seed embryo is surrounded and fed by an endosperm, a tissue equivalent to the placenta in mammals. Endosperm development is as important as the embryo for the generation of viable seeds. In sexual reproduction, the endosperm is triploid as the result of the fusion of the female diploid central cell with one haploid sperm cell. In most apomictic plants, the embryo develops without fertilization (parthenogenesis), but for endosperm (see above), the central cell must be pollinated and fertilized, a process called pseudogamy (Hojsgaard and Hörandl 2019; Nogler 1984). Autonomous development of endosperm is also reported in a few apomicts that do not need fertilization (Koltunow 1994; Koltunow and Grossniklaus 2003).

## Engineering apomixis step 1: apomeiosis

Despite being present in many species, apomixis is absent in major crops. In the last decade, much progress has been made in engineering de novo apomixis by tinkering with the process of sexual reproduction. The first crucial step is to induce apomeiosis, the modification of meiosis into a mitotic-like division. Three crucial events distinguish meiosis from mitosis: (i) recombination in prophase of meiosis I, (ii) co-segregation of sister chromatids at meiosis I, and (iii) a second round of division. Abolishing each of these differences with appropriate mutations can turn meiosis into mitosis as a strategy termed MiMe (Mitosis-instead-of-Meiosis, Table 2, Fig. 1A) (d’Erfurth et al. 2009), the molecular basis of which follows from functional study of each respective meiotic event.

**Table 2:** Genes used in synthetic apomeiosis

	Mutant name	Role of gene	Desired phenotype	Arabidopsis thaliana gene ID	Zea maize, Rice	Penetrance	Functionally characterized in	References
	*spo11-1*	Meiosis-specific DNA DSB formation	Abolishment of recombination	At3G13170	Os03g54091	Complete	At, Os	Fayos et al. (2020), Grelon et al. (2001), Yu et al. (2010)
	*spo11-2*	Meiosis-specific DNA DSB formation	Abolishment of recombination	At1G63990	Os08g06050	Complete	At, Os, Ta	Benyahya et al. (2020), Fayos et al. (2020), Stacey et al. (2006)
	*mtopVIB*	Meiosis-specific DNA DSB formation	Abolishment of recombination	At1G60460	Os06g49450	Complete	At, Os, Zm	Vrielynck et al. (2016), Xue et al. (2016)
	*prd1*	Meiosis-specific DNA DSB formation	Abolishment of recombination	At4G14180	Os04g28020	Complete	At, Os	De Muyt et al. (2007), Shi et al. (2021)
	*prd2*	Meiosis-specific DNA DSB formation	Abolishment of recombination	At5G57880	Os08g44180	Complete	At	De Muyt et al. (2009)
	*pair1/prd3*	Meiosis-specific DNA DSB formation	Abolishment of recombination	At1G01690	Os03g01590	Complete	At, Os	De Muyt et al. (2009), Nonomura et al. (2004)
	*dfo*	Meiosis-specific DNA DSB formation	Abolishment of recombination	At1G07060	Os12g01540	Complete	At	Zhang et al. (2012)
	*rec8*	Meiosis-specific cohesin Subunit	Loss of monopolar orientation at meiosis I	At5005490	Os05g50410	Complete	At, Os, Zm	Chelysheva et al. (2005), Golubovskaya et al. (2006), Shao et al. (2011)
	*osd1*	Inhibitor of APC/C	Skip Meiosis II division	At3G57860	Os02g37850	Very high frequency	At, Os	d’Erfurth et al. (2009), Mieulet et al. (2016)
	*tam/cyca1;2*	Cyclin A1;2	Skip Meiosis II division	At1G77390	Os01g13260, Os01g13229, Os12g20324, Os05g14730	High	At	d’Erfurth et al. (2010)
	*tdm -p17*	APC/C regulators	Skip Meiosis II division	At4G20900	Os08g03620	High and dominant	At	Cifuentes et al. (2016), Glover et al. (1998)
MiMe	*spo11-1 rec8 osd1*	Generation of apomeiosis	Clonal gametes	See above	See above	Very high frequency	At	d’Erfurth et al. (2009)
MiMe	*pair1 rec8 osd1*	Generation of apomeiosis	Clonal gametes	See above	See above	Very high frequency	Os, At	Khanday et al. (2019), Mieulet et al. (2016), Wang et al. (2019)
MiMe	*prd1 rec8 osd1*	Generation of apomeiosis	Clonal gametes	See above	See above	Very high frequency	At	Mieulet et al. (2016)
MiMe	*prd2 rec8 osd1*	Generation of apomeiosis	Clonal gametes	See above	See above	Very high frequency	At	Mieulet et al. (2016)
MiMe	*spo11-1 rec8 tam*	Generation of apomeiosis	Clonal gametes	See above	See above	High	At	d’Erfurth et al. (2010)
MiMe	*spo11-1 rec8 tdm p17*	Generation of apomeiosis	Clonal gametes	See above	See above	High	At	Cifuentes et al. (2016)
	*dyad /swi1/am1*	Generation of apomeiosis	Clonal gametes	At5G51330	Os03g44760, GRMZM5G883855	Low frequency	At, Os, Zm	Golubovskaya et al. (1997), Pawlowski et al. (2009), Ravi et al. (2008)
	*ago104/dnr4/ago9*	Generation of apomeiosis	Diplod/clonal gametes	AT5G21150	Os04g06770, GRMZM2G141818	Low frequency	At, Zm	Olmedo-Monfil et al. (2010), Singh et al. (2011)
	*nrf4*	Generation of apomeiosis	Diplod/clonal gametes	–	Os07g46290, GRMZM2G148133	Low frequency	Zm	W et al. (2016) WO Patent 2016/179522

**(i) Recombination** in meiosis is initiated with the formation of DNA double-strand breaks (DSB), and a series of proteins catalyzing this step are conserved from yeast to humans and plants. The SPO11 (DNA topoisomerase) complex is a tetramer composed of two copies of TOPOVIB and one copy each of SPO11-1 and SPO11-2 (Grelon et al. 2001; Hartung et al. 2007; Stacey et al. 2006; Vrielynck et al. 2016) and acts as a major player to catalyze DSB formation. In addition to the SPO11 complex, three more proteins – (Putative Recombination initiation Defect) PRD1, PRD2 and PRD3/PAIR1 – are also required for DSB formation (De Muyt et al. 2007, 2009; Nonomura et al. 2004). Mutation of any of these genes completely eliminates the recombination process. The *spo11-1*, *prd1*, *pdr2* and *prd3/pair1* were each shown to be efficient to generate MiMe (d’Erfurth et al. 2009; Mieulet et al. 2016) as would certainly any mutation that abolishes recombination (e.g*., spo11-2* and *topoVIb*). An additional gene, *DFO,* is essential for formation of DSB in Arabidopsis, but its homologs have not been functionally analyzed in other plant species (Zhang et al. 2012). Additional genes, like SDS (Wu et al. 2015), PCH2/OsCRC1 (Miao et al. 2013), and P31^comet^ (Ji et al. 2016), are involved in the initiation of meiotic recombination in rice but play a downstream role in the recombination process in Arabidopsis (Balboni et al. 2020; Lambing et al. 2015) (De Muyt et al. 2009; Wijeratne et al. 2006). Many other genes are needed at later stages of recombination (Mercier et al. 2015; Wang and Copenhaver 2018), but their mutation does not completely abolish recombination, making them not suitable for engineering apomeiosis with the MiMe concept.

(ii) **Monopolar orientation** of sister chromatids in meiosis is the second key difference between meiosis and mitosis. In mitosis, when two newly synthesized sister chromatids align at metaphase, their kinetochore (a protein complex at centromeres where the spindle binds) orients toward opposite poles, and as a result, sister chromatids move to the opposite poles. The opposite orientation of the sister kinetochores is called bipolar orientation. In contrast to mitosis, in meiosis each homologous chromosome consists of two sister chromatids that co-orient toward the same pole, a scenario known as monopolar orientation. The monopolar orientation at meiosis persists even in the absence of COs (e.g., *spo11-1* mutants; Grelon et al. 2001), and sister chromatids segregate together. As they lack crossovers, the homologous chromosomes segregate randomly at meiosis I, leading to aneuploidy. Loss of monopolar orientation of the kinetochores, together with the complete abolishment of COs, allows equational segregation of chromatids at the first meiotic division, mimicking mitosis (d’Erfurth et al. 2009; Mieulet et al. 2016). In plants, the monopolar orientation of sister kinetochores at metaphase I relies on the cohesin complex (Chelysheva et al. 2005). Cohesin is a four-protein complex that forms a ring-like structure and keeps sister chromatids together after replication (Anderson et al. 2002; Gruber et al. 2003; Haering et al. 2002; Nasmyth 2002). In plants, one cohesion subunit has a meiosis-specific variant, REC8 (Bai et al. 1999; Bhatt et al. 1999). Combining the *rec8* mutation with abolition of recombination results in a mitotic-like division at meiosis I with separation of the sister chromatids (Fig. 1A). However, the meiocyte then undergoes a second round of division and free chromatids segregate randomly, causing sterility (Chelysheva et al. 2005; d’Erfurth et al. 2009). Mutations of the other cohesin subunits are embryonic-lethal (Lam et al. 2005; Liu et al. 2002; Tzafrir et al. 2002) and thus cannot be used to generate apomeiosis. The cohesin subunit SCC3 has been shown to be crucial for monopolar orientation, as a weak mutant allele of SCC3 induces loss of monopolar orientation at meiosis I, but also displays somatic developmental defects (Chelysheva et al. 2005). Similarly, a kinetochore protein, MIS12, is essential in plants, but was shown by RNAi to be involved in monopolar orientation in maize (Li and Dawe 2009; Sato et al. 2005). Some meiosis-specific proteins, such as Mam1 in *Saccharomyces cerevisiae*, Moa1 in *Schizosaccharomyces pombe*, and Meikin in mammals enforce monopolar orientation, but equivalent proteins in plants have yet to be identified (Kim et al. 2015; Toth et al. 2000; Yokobayashi and Watanabe 2005). If such function exists in plants, they could be alternatives to REC8 to engineer apomeiosis.

**(iii) Two rounds of division** The two consecutive rounds of division during meiosis constitute a third key difference from mitosis. In *spo11 rec8*, the first division mimics a mitotic division (see above), but the second division still occurs, leading to meiotic catastrophe. A solution to this problem was presented by the identification of the Arabidopsis mutant *osd1* (*omission of second division1*) (d’Erfurth et al. 2009). OSD1 is an APC/C (anaphase promoting complex/cyclosome) regulator, and its mutation causes skipping of meiosis II and consequently the generation of diploid spores and gametes. The frequency of diploid gametes in *osd1* is 100% for males and 85% for females (d’Erfurth et al. 2009). The diploid gametes of *osd1* are recombinant because recombination and homologous chromosome segregation still occur at meiosis I (d’Erfurth et al. 2009).

UVI4 is a paralog of Arabidopsis OSD1 that is also an APC/C inhibitor (Cromer et al. 2012; Heyman et al. 2011; Iwata et al. 2011; Van Leene et al. 2010). In *uvi4,* meiosis is normal but high rates of endoreduplication occur in somatic tissue. The two genes have some overlapping roles as double mutants are embryonic-lethal, indicating that OSD1 and UVI4 are crucial for somatic cell division (Cromer et al. 2012; Iwata et al. 2011). In brassicas, to which Arabidopsis belongs, the OSD1 and UVI4 orthologs are easily recognized as arising from a whole-genome duplication event that occurred at the root of this clade (Lloyd et al. 2014; Mieulet et al. 2016). Beyond the Brassicaceae, OSD1/UVI4 is typically represented by a single gene except in recent polyploids. In the clade of Poaceae, an ancient independent duplication produced two gene families (Mieulet et al. 2016). One member of the duplication was functionally characterized in rice to recapitulate the Arabidopsis *osd1* phenotype, designated OsOSD1 (Mieulet et al. 2016). The frequency of diploid gamete formation is 100% in males and ~ 90% in females, similar to Arabidopsis (Mieulet et al. 2016). The second gene of OSD1/UVI 4 in rice has not yet been characterized, and based on Arabidopsis, it can be speculated that the double mutant would be lethal. Although OsOSD1 is phylogenetically distant from Arabidopsis OSD1 and UVI4, it seems to have acquired a meiotic role via convergent evolution. Therefore, OSD1 homologs can be considered promising candidate genes in Brassicaceae and Poaceae for skipping meiosis II and establishing MiMe. However, many plant species outside of the Brassicaceae and Poaceae families, like tomato and melon (Solanaceae or Cucurbitaceae), contain only one copy of either OSD1 or UVI4. Therefore, it is quite possible that its mutation would be lethal. As such, an alternative for OSD1 would be helpful for making MiMe work beyond Brassicaceae and Poaceae.

In Arabidopsis, a mutant called TARDY ASYNCHRONOUS MEIOSIS (TAM)/Cyclin CYCA1;2 was found that also skips meiosis II and results in diploid gametes similarly to *osd1*, as it is needed for entry into meiosis II (Cromer et al. 2012; d’Erfurth et al. 2010). However, the frequency of diploid gamete formation in *tam1* is nearly 95% in males but only ~ 40% in females, which is weaker compared to *osd1* (Cromer et al. 2012) and represents a limitation as the unreduced female egg is the main target cell for apomixis generation. Interestingly, combining *osd1* and *tam1* increased the frequency of diploid gametes in females up to 99%. However, males are quasi-sterile because male meiocytes are arrested at prophase I, which may pose a problem for creating apomixis with regard to endosperm formation (Cromer et al. 2012). Although the *TAM1/CyclinA1* gene family is well conserved at the sequence level, it remains to be functionally characterized beyond Arabidopsis.

Another alternative to OSD1 is THREE DIVISION MUTANT1 (TDM1), although it plays an opposite role to TAM1 or OSD1; *tdm1* knockout causes an aberrant third meiotic division after normal meiosis I and II (Cromer et al. 2012; Glover et al. 1998; Ross et al. 1997). TDM1 is proposed to be a meiosis-specific APC/C component that stimulates APC/C for meiotic exit immediately following meiosis II (Cifuentes et al. 2016). It has been also proposed that TDM1-containing P-bodies reduce the expression of meiotic transcripts to ease the switch of cell fates to post-meiotic gametophyte development (Cairo et al. 2022). CYCA1;2/TAM negatively regulates TDM through phosphorylation, as mutation of a phosphorylation site (threonine 16 on Arabidopsis TDM1) dominantly provokes the *tam1* phenotype, i.e., the skipping of meiosis II, leading to diploid gamete production (Cifuentes et al. 2016). Therefore, a dominant mutation of T*DM1* can be used to substitute *osd1* or *tam* for generation of MiMe (Cifuentes et al. 2016). The TDM1 protein sequence, together with its consensus phosphorylation site, is conserved across angiosperms but has yet to be functionally characterized beyond Arabidopsis.

Two more mutants, *Atps1* (*Arabidopsis thaliana* parallel spindle I) and *Jason*, produce diploid gametes due to the fusion of meiotic II spindles (d’Erfurth et al. 2008; De Storme and Geelen 2011). The frequency of diploid gametes transmitted to the next generation is less than 30% in both mutants and is restricted to males (Crismani et al. 2013; d’Erfurth et al. 2008; De Storme and Geelen 2011). Therefore, Jason and Atps1 appear as less viable candidates for MiMe generation.

**(iv) Combining the three turns meiosis into mitosis** MiMe, a highly efficient apomeiosis, was created in Arabidopsis by combining one mutant for each function: (i) *spo11* to prevent recombination, (ii) *rec8* to prevent monopolar orientation, and (iii) *osd1* to skip meiosis II (d’Erfurth et al. 2009). MiMe-2 was also successfully introduced to Arabidopsis by replacing *osd1* with *tam1*, but 10–15% of the female gametes were aneuploid, most likely due to the leakiness of *tam1* (Cromer et al. 2012). By comparing the original MiMe to MiMe-2, it appears that OSD1 would be the first choice for generating MiMe, but it is possible that the penetrance of the MiMe phenotype may vary in different species. The rice MiMe was also created in the same way, but by using *prd3/pair1* instead of *spo11* (Mieulet et al. 2016). MiMe gametes are clones of maternal cells; thus, ploidy is doubled in the subsequent generations due to the fusion of diploid gametes.

## Alternatives to MiMe for engineered apomeiosis

In Arabidopsis, DYAD/SWI1 is a crucial protein for meiosis that has been shown to protect the cohesin complex during meiotic prophase (Agashe et al. 2002; Mercier et al. 2001, 2003; Yang et al. 2019). Mutation of this single gene results in 50% of progeny deriving from apomeiosis, but it is a quasi-sterile mutant, limiting applied perspectives (Marimuthu et al. 2011; Ravi et al. 2008). The AMEIOTIC mutant, which is a homolog of DYAD/SWI1, was studied in rice and maize, but displayed high sterility (Che et al. 2011; Golubovskaya et al. 1997; Pawlowski et al. 2009). In maize, the *nonreduction in female 4 (nrf4)* mutant forms diploid female gametes, but only about 5% of them are clonal (Fox et al. 2016). Some epigenetic regulators have also been found to have a role in the control of meiosis in plants. The maize DNA methylation mutants *dmt102* and *dmt103* can induce unreduced gamete formation (Garcia-Aguilar et al. 2010). The *Dnr4* ortholog in maize is known as *AGO104*; its mutation causes apomeiosis and produces diploid gametes with a 40–70% frequency in females (Singh et al. 2011). Similarly, in Arabidopsis ARGONAUTE9 (AGO9) regulates female gamete formation through SUPPRESSOR OF GENE SILENCING3 (SGS3) and RNA-DEPENDENT RNA POLYMERASE6 (RDR6); mutation of any of these three genes leads to the formation of multiple gametic cells (Olmedo-Monfil et al. 2010). Thus, despite requiring three distinct mutations, it appears that MiMe remains the most efficient way to engineer apomeiosis.

## Engineering apomixis step 2: parthenogenesis

### A fertilization checkpoint before embryogenesis

While turning off key meiotic genes is sufficient to engineer apomeiosis, the doubling of ploidy with each generation means that MiMe alone cannot produce clonal progeny. Fertilization of diploid gametes is unaffected, and thus, MiMe mutants lack a crucial component of apomixis: embryogenesis without fertilization. As previously described, double fertilization is common to most plants and results in the formation of a zygote and an endosperm progenitor cell, the former developing into an embryo and the latter required for the proper development of the embryo (West and Harada 1993). A wide range of plant taxa have been shown to possess a trait known as parthenogenesis, in which embryos spontaneously form without fertilization, giving rise to haploid or diploid progeny (Bierzychudek 1985; Nygren 1954). In the quest to engineer parthenogenesis, great strides have been made in recent decades in elucidating the genetics controlling the transition from an unfertilized ovule to a developing embryo (Table 3). Numerous studies have implicated single dominant loci in the control of parthenogenesis in gametophytic apomicts and have demonstrated that the formation of a diploid egg cell (apospory) and the formation of a diploid embryo (parthenogenesis) are controlled separately (Albertini et al. 2001; Ogawa et al. 2013; van Dijk et al. 1999). A single dominant gene, *PsASGR-BBML* (*P. squamulatum* apospory-specific genomic region BABY BOOM-like), was found to segregate with the occurrence of apospory in the grass species Pennisetum and was shown to generate diploid offspring in sexual pearl millet tetraploids (Conner et al. 2015). Transgenic lines in rice and maize carrying *PsASGR-BBML* using either a native *P. squamulatum* promoter or a DD45 promoter conferring egg cell-specific expression saw high rates of haploid embryo formation and haploid plant recovery (Conner et al. 2017), supporting the transferability and potential application of BBM-like genes in monocot crops, although whether specific expression and/or a specific function of the protein was required for haploid induction remained unclear. BABY BOOM was initially identified in *Brassica napus* as an APELATA2/ETHYLENE RESPONSIVE FACTOR (AP2/ERF) domain-containing gene preferentially expressed in developing embryos and able to induce somatic embryo structures when ectopically expressed (Boutilier et al. 2002), suggestive of a role in morphogenesis and the regulation of embryo development. Analysis of a BBM-like gene in rice, *OsBBM1*, found that it can similarly induce somatic embryos, but crucially is exclusively expressed in the male genome prior to fertilization (Khanday et al. 2019). Ectopic expression of wild-type *OsBBM1* under the egg cell-specific DD45 promoter induced the formation of haploid embryos, supporting a model in which expression from the male gamete during fertilization triggers BBM1 expression in the embryo (Khanday et al. 2019, Fig. 1B). These findings suggest that BBM1 acts as a male trigger for embryogenesis and shows that the fertilization checkpoint can be overridden by BBM1 misexpression in the female genome to induce parthenogenesis.

**Table 3:** Genes used in synthetic parthenogenesis

Gene name	Aim	Functionally characterized in	Arabidopsis thaliana	Rice	Penetrance in species	Applied in	References
*PsASGR-BBML*	Parthenogenesis	Pennisetum squamulatum	AT5G17430	Os11g19060	100%	Pearl millet, rice, maize, tobacco	Conner et al. (2015, (2017), Zhang et al. (2020)
*OsBBM1*	Parthenogenesis	Rice	AT5G17430	Os11g19060	5–29%	Not tested	Khanday et al. (2019), Vernet et al. (2022)
*ToPAR*	Parthenogenesis	Dandelion	AT4G35610/AT4G35700	Not identified	100%	Lettuce	Underwood et al. (2022)
*Modified CENH3*	Haploid induction	Arabidopsis	AT1G01370	Not identified	1–34%	Maize, wheat, melon, cucumber, watermelon, rice, tomato	Kelliher et al. (2016), Kuppu et al. (2015, (2020), Ravi and Chan (2010), Wang et al. (2021) patents 2017/081011,2017/200386
*ZmMATRILINEAL*	Haploid induction	Maize	Not identified	Os03g27610	1–6%	Rice, wheat, foxtail, millet	Gilles et al. (2017), Kelliher et al. (2017), Liu et al. (2017), Liu et al. (2020), Yao et al. (2018)
*ZmDMP*	Haploid induction	Maize	AT1G09157/AT5G39650	Not identified	0.1–0.3%	Arabidopsis, tomato, tobacco, rapeseed, Medicago	Wang, Xia, et al. (2022a, b), Zhong et al. (2019, (2020)

The perhaps best-described natural apomict is common dandelion (*Taraxacum officinale*); genetic segregation experiments from the last two decades in dandelion and its close Asteraceae relative hawkweed (*Hieracium*) implicate distinct loci in controlling different aspects of apomixis. In dandelion, a locus controlling diplospory (DIP) and a separate parthenogenesis (PAR) locus were identified (van Dijk et al. 1999; van Dijk and Bakx-Schotman 2004; Vijverberg et al. 2010), with a complex third component tightly linked to the PAR locus conferring autonomous endosperm (AutE) necessary for apomixis in hawkweed and dandelion (Ogawa et al. 2013; Van Dijk et al. 2020). In aposporous hawkweed, a separate loss-of-apomeiosis (LOA) locus was characterized (Catanach et al. 2006). Most recently, deletion mapping on the basis of a loss-of-parthenogenesis phenotype and clustered regularly interspaced short palindromic repeats-based mutagenesis (CRISPR-Cas9) screening of candidate genes was used to refine the PAR locus and implicate a single dominant gene in dandelion parthenogenesis (Underwood et al. 2022). The authors found that a large transposable element (miniature inverted-repeat transposable element, or MITE) inserted within the PAR gene promoter is specific to the apomictic allele and causal for parthenogenesis. The authors next transformed parthenogenesis-deficient mutants with the MITE promoter fused to a lettuce PAR homolog, finding that the MITE-containing promoter is able to restore parthenogenesis. Fusions of the dandelion-derived PAR gene with the AtEC1 egg cell promoter could similarly complement loss-of-parthenogenesis mutants in dandelion and further induce parthenogenesis in sexual lettuce, suggesting a common mechanism in both species. These findings and the presence of an EAR-repressive motif led the authors to propose that PAR may act as a repressor of an unidentified gene suppressing embryogenesis in the egg cell; the inserted transposon could allow for PAR expression within the egg cell and trigger embryogenesis independent of sperm-contributed PAR (Underwood et al. 2022, Fig. 1B). Thus, in a natural apomict, misexpression of a dominant gene in the female gametophyte can drive parthenogenesis in a similar manner to egg cell-specific expression of BBM1. While the contribution of autonomous endosperm and diplospory still appear to be required for apomixis in dandelion and await functional characterization, PAR shows immediate promise for the engineering of parthenogenesis in dicot crops.

### Haploid induction to bypass fertilization

An alternative approach to inducing apomixis lies in the pairing of apomeiosis with directed genome elimination. Preventing one of the parental genomes from contributing to fertilization, often through single loss-of-function mutations, has been shown to effectively trigger haploid induction. Mutations within the centromere-specific histone CENH3 in Arabidopsis were shown to eliminate the altered parent genome contribution upon crossing to a wild-type receiver, allowing for the production of maternal or paternal haploids (Ravi and Chan 2010, Fig. 1C). Haploid induction via CENH3 has to date been successfully implemented in maize and wheat (Karimi-Ashtiyani et al. 2015; Kelliher et al. 2016; Lv et al. 2020; Wang et al. 2021), and has been further shown to improve haploid induction rates when paired with the maize Stock6 inducer (Meng et al. 2022). While haploid induction rates of up to 45% have been shown in Arabidopsis using CENH3-mediated genome elimination (Ravi and Chan 2010), induction rates have not exceeded 5–7% in maize and wheat (Lv et al. 2020; Wang et al. 2021), well below commercially used inducer lines. One advantage in both crop systems described, however, is the ability to produce both maternal and paternal haploids, and pairing with other haploid induction strategies may be effective in increasing induction rates.

An alternative mechanism that similarly relies upon the mechanism of uniparental genome elimination was identified in a loss-of-function allele of the MATRILINEAL gene (*ZmMTL/PLA1/NLD*). Genetic mapping by three independent research groups of maize Stock 6, a widely used mutant crucial in haploid induction, implicated a frame-shift mutation in the phospholipase *ZmMTL* as causal for triggering haploid induction (Gilles et al. 2017; Kelliher et al. 2017; Liu et al. 2017). *ZmMTL/PLA1/NLD* was found to be exclusively expressed in pollen and is likely to encode a membrane-localized phospholipase gene with pleiotropic effects on pollen development and gene expression (Gilles et al. 2017; Kelliher et al. 2017). Mutations in *MTL/PLA1/NLD* have to date shown promise in haploid induction in elite cultivars of the major crops maize, rice, and wheat (Kelliher et al. 2019; Liu et al. 2020; Wang et al. 2019; Yao et al. 2018; Fig. 1C). Mutation of a *ZmMTL/PLA1/NLD* homolog within the Phospholipase D gene class, *ZmPLD3*, was also shown to produce similar levels of haploids (Li et al. 2021). Single-cell sequencing revealed that high levels of aneuploidy persist in Stock-6 derived pollen when compared to post-meiotic tetrads, suggesting that the underlying basis of haploid induction in loss-of-function *mtl* is driven by chromosome fragmentation (Li et al. 2017). Recent studies in wheat and maize implicated reactive oxygen species (ROS) in haploid induction driven by *mtl/pla1/nld* (Jiang et al. 2022; Sun et al. 2022) and showed that a lipid imbalance within *mtl/pla1/nld* pollen results in a ROS burst that fragments paternal DNA (Jiang et al. 2022). CRISPR mutagenesis of maize genes involved in ROS production identified a peroxidase gene, *ZmPOD65*, that induces haploids by utilizing the same mechanism, and it was further shown that simply treating pollen with ROS-inducing agents can produce haploids in various backgrounds (Jiang et al. 2022). Whether directly inducing ROS formation may prove useful in generating haploids efficiently and in other species is not yet clear.

While *MTL/PLA1/NLD* appears to be present exclusively in monocots, mapping of the *qhir1* locus present in the maize haploid inducer line CAU5 uncovered a novel loss-of-function allele mutation within *ZmDMP*, which encodes a putative DUF679 membrane protein (Zhong et al. 2019). *ZmDMP* was found to be preferentially expressed in pollen and to be notably conserved among both monocots and dicots. Knockout mutants generated in *ZmDMP* alone saw a haploid induction rate of up to 0.3% but were shown to significantly increase haploid induction in the *mtl/pla1/nld* background to a maximum of 7% (Zhong et al. 2019). Mutation of *ZmDMP*-like genes in Arabidopsis confirmed its functional conservation within dicots (Zhong et al. 2020), paving the way for successful haploid induction in legumes and brassicas (Wang et al. 2022a, b; Zhao et al. 2022). DMP-like genes thus harbor the potential of allowing higher haploid induction rates in a range of monocots and a loss-of-function strategy for engineered parthenogenesis in dicots. The mechanism of DMP-like haploid induction remains to be clarified but could provide novel means of haploid production as seen with *ZmMTL/PLA1/NLD* and ROS induction.

## Applied apomixis

### Engineered apomixis in principle and in hybrid crops

The above sections highlight significant leaps in our understanding of apomeiosis and parthenogenesis and offer means of their application, which in tandem are required to engineer apomixis in the pursuit of clonal seeds. Combining MiMe-driven apomeiosis with the described strategies to engineer parthenogenesis can produce clonal diploid gametes that develop into synthetic apomicts (Fig. 1D). Pairing of CENH3-mediated genome elimination (GEM) with MiMe to bypass both diploid embryo formation and meiotic shuffling was first described in Arabidopsis as a means to obtain clonal seeds (Marimuthu et al. 2011). Over a third of the resultant progeny of GEM and MiMe crosses were diploid and identical to the maternal parent; repeated crossing with GEM could further fix the genetic information in subsequent generations (Marimuthu et al. 2011). Despite low seed viability and the requirement for repeated crossing, this work demonstrated that synthetic clonal propagation is feasible and yields products that are identical to those of natural apomixis. Following the finding that MiMe could be extended to rice (Mieulet et al. 2016), synthetic and autonomous clonal reproduction was first demonstrated in a major monocot crop using either BBM1- or MTL-based parthenogenesis induction (Khanday et al. 2019; Wang et al. 2019). By skipping meiosis using MiMe and targeting *OsBBM1* expression to the egg cell, heterozygous SNPs present in the maternal parent were maintained for more than two generations (Khanday et al. 2019). Apomictic frequencies of up to 29% were obtained; the authors reasoned that incomplete parthenogenesis rather than variation in endosperm ploidy limits this frequency, as no defects in seed size or development were observed. It was recently demonstrated that improvements in parthenogenetic induction can in fact significantly increase the frequency of clonal progeny obtained, notably in a commercial F1 hybrid (Vernet et al. 2022). This work provides further support that engineering autonomous endosperm may not be necessary for efficient synthetic apomixis. Apomictic frequencies of greater than 95% could be maintained for at least three generations when a single DD45-BBM1-MiMe cassette was employed, well above previous frequencies (Khanday et al. 2019). While the source of the increased efficiency is unclear, one hypothesis proposes that the all-in-one construct enhances expression of BBM1 and underlies the difference. Deficiencies in fertility may be attributed to incomplete penetrance of *osd1* loss of function, resulting in continued production of a low level of haploid gametes following female meiosis (Mieulet et al. 2016). This is functionally distinct from parthenogenesis and the problem could potentially be addressed by using alternative regulators of meiotic progression (d’Erfurth et al. 2010). One piece of evidence suggests that egg cell-directed OsBBM1 expression can induce the development of a second embryo from egg cell-adjacent synergid cells, the consequences of which, however, are unclear (Junhao et al. 2022). The alternative strategy pairs null *OsMTL* alleles with MiMe in a single gene editing cassette to produce clonal progeny directly in a hybrid variety (Wang et al. 2019), although with significant reductions in fertility compared to BBM1 strategies, a known consequence of *OsMTL*-driven haploid induction (Yao et al. 2018). It was recently proposed that incomplete genome elimination by *osmtl* may be responsible for observed losses in fertility (Liu et al. 2022). Despite this limitation, the maintenance of heterosis in engineered apomict crops has been reported using both MTL and BBM1 strategies (Liu et al. 2022; Vernet et al. 2022), supplementing work in *Hieracium* proving that natural apomicts can indeed transmit complex phenotypes derived from hybrids (Sailer et al. 2016). Reduced seed viability remains a hurdle and appears to arise from multiple sources, but strategies that mitigate against this problem exist. The implementation of synthetic apomixis in other economically important crops has yet to be reported, though the conservation of key meiotic genes (Hyde et al. 2022; Ma et al. 2018) as well as BBM, MTL, and DMP-like genes (Chen et al. 2022; Liu et al. 2020; Wang, Xia, et al. 2022a, b) offers promise. Further worth considering is the recent clarification of the locus controlling nucellar or adventitious embryony in citrus (Wang et al. 2017), a defining feature of sporophytic apomixis. Comparative genomics identified the co-segregation of citrus polyembryony with a MITE insertion in the promoter of a candidate gene termed CitRWP, possessing a domain known to influence embryogenesis (Waki et al. 2011). While polyembryony is observed in natural gametophytic apomicts, further functional validation is required and the persistence of both sexual and asexual embryos may pose problems for efficient engineered apomixis. The current body of work demonstrates that high levels of clonal progeny in crops can be obtained by sidestepping meiosis and excluding the contribution of one of the parental genomes. The combined apomeiosis-parthenogenesis provides a strategy that is fully penetrant and able to propagate hybrid vigor, though limitations remain in terms of fertility.

### Outlook for engineered apomixis and potential roadblocks

It is now clear that by fine-tuning a few key regulators of meiosis and embryogenesis, one can engineer apomixis within several major crops. While exciting, the acceptance of such technologies face significant hurdles with regard to the end consumer and the legislation that regulates their use (Batalha et al. 2021; Turnbull et al. 2021). The methodologies presented in this review are termed and regulated as two separate technologies in many countries, namely genetic engineering or modification (GM) and genome editing (GE). While the former is subject to extensive regulation by many governing bodies, the latter has been granted exemption by some governments (S. M. Schmidt et al. 2020), acknowledging the fact that GE does not introduce large pieces of foreign DNA and is thus similar in outcome to the product of conventional and mutagenesis breeding (Turnbull et al. 2021). This discrepancy has had a profound influence on the commercialization of CRISPR-based genome-edited products (Martin-Laffon et al. 2019). Despite continued review of such policies (Friedrichs et al. 2019) and proposals to integrate GE technologies (Huang et al. 2016), a mismatch nonetheless exists between the pace of acceptance and the acceleration of their development. Utilization of MiMe and MTL mutations to engineer apomeiosis and parthenogenesis may fall in the category of GE, as they require simple edits through CRISPR-based approaches. Misexpression of BBM1/PAR in the developing embryo, however, is dependent upon the stable introduction of recombinant DNA, namely alternative promoter sequences, and thus has the potential to see greater regulatory pushback regardless of whether these sequences remain plant-derived.

A number of strategies have been reported in recent years that aim to assuage some legislative and technical concerns related to genome editing. While the product of CRISPR-mediated genome editing does not retain large pieces of inserted foreign DNA, introducing edits often requires stable expression of a CRISPR/Cas9 transgene cassette through transformation. Genetic segregation can remove the transgene in subsequent generations, but this approach would not be possible in a clonally reproducing synthetic apomict. One tactic couples the transformation of a CRISPR/Cas9 cassette with the expression of two genes that trigger cell death during embryo development and within the male gametophyte, termed TKC (Transgene Killer CRISPR) (He et al. 2018). Expression of the toxic *BARNASE* gene during rice embryo development and rice *CMS2* conferring male sterility, together with a genome editing construct, can induce cell death in reproductive tissue containing transgenes. This approach ensures that any seeds containing the transformed construct do not develop and precludes the need for transgene removal by segregation. Alternatively, a method that does not require insertion of foreign DNA introduces CRISPR/Cas9-bundled ribonucleoproteins (RNPs) to direct editing. Several reports have demonstrated success in delivering RNPs into protoplasts, embryos, or zygotes of crop species to confer entirely DNA-free genome editing (Liang et al. 2017; Toda et al. 2019; Woo et al. 2015). Both TKC and RNP editing methods have the added benefit of reducing possible off-targets during editing, a concern common to both regulators and researchers (Hahn and Nekrasov 2019; S. M. Schmidt et al. 2020). Recent work also demonstrated that CRISPR cassettes can be virally delivered to induce heritable mutations in a model system (Ma et al. 2020), opening the door to further transgene-free options. While the above represent methods to resolve potential GE concerns, incorporation of stable transgenes via GM is still necessary in several strategies to engineer apomixis and remains a possible roadblock for product development.

Following its acceptance by regulators, questions still remain as to how exactly engineered apomixis can fit into modern breeding schemes. Apomicts derived from dominant mutations have been suggested to act as pollen donors for crossing to sexual elite varieties (van Dijk et al. 2016), a strategy that could greatly reduce the time and resource-intensive breeding cycles and one that remains compatible with synthetic apomixis strategies described above. Alternatively, direct manipulation of elite varieties could further shorten and simplify the breeding cycle, provided that transformation remains feasible. An additional concern lies in the intellectual property rights surrounding apomixis technology; while frameworks are currently limited, a proposal put forth by apomixis researchers in 1998 stressed the importance of maintaining equitable access to such technologies for the benefit of global food security (Grossniklaus et al. 1998). Lastly, the emergence of synthetic apomicts will undoubtedly influence the economics of seed production by allowing hybrids to self-propagate similar to current elite non-hybrid pure lines. In the context of a seed market that is evenly split between public institutions, private industry, and farmer seed-saving, it could have a net positive effect for each group. It has been proposed that savings to both growers and industries could reach upwards of billions of dollars per year (Spillane et al. 2004), while simultaneously facilitating true seed production, diversifying hybrid breeding, and consequently improving adaptability in changing climates.
